# Examining the influence of global smoking prevalence on stroke mortality: insights from 27 countries across income strata

**DOI:** 10.1186/s12889-024-18250-1

**Published:** 2024-03-19

**Authors:** Isuri Abeysekera, Roshinie De Silva, Disuri Silva, Lakindu Piumika, Ruwan Jayathilaka, Lochana Rajamanthri

**Affiliations:** Department of Information Management, SLIIT Business School, Sri Lanka Institute of Information Technology, New Kandy Road, Malabe, Sri Lanka

**Keywords:** Global smoking prevalence, Stroke death rates, Stroke incidence, Global death rate, Income groups

## Abstract

**Background:**

This study investigates the influence of Global Smoking Prevalence (GSP) on Stroke Death Rates (SDR) across 27 countries categorized into High-Income Countries (HIC), Upper Middle-Income Countries (UMIC), Lower Middle-Income Countries (LMIC), and Low-Income Countries (LIC).

**Methods:**

Analysing data from two distinct periods (1990–1999 and 2010–2019), countries exhibiting an increased SDR were selected. The study uses a polynomial regression model, treating income groups as cross-sectional and years as time series data.

**Results:**

Results from the regression model reveal that 17 countries observed a significant impact of GSP on SDR, with only Turkey, Solomon Islands, and Timor-Leste resulting in negative values. However, the study emphasises that out of all 27 countries, the highest occurrence of the impact of GSP on SDR has been reported in the LMIC stratum for the period under review.

**Conclusion:**

It is evident that GSP affects the risk of incidence of stroke death, specifically in the LMIC stratum. Furthermore, it has been identified that GSP is a major preventable risk factor affecting global mortality. To mitigate the risk of stroke death attributable to smoking prevalence, necessary preventive steps should be adopted to encourage smoking cessation, and essential policies should be implemented to reduce the burden of SDR.

**Supplementary Information:**

The online version contains supplementary material available at 10.1186/s12889-024-18250-1.

## Introduction

Smoking is widely regarded as one of the major health hazards worldwide, leading to millions of people suffering from poor health conditions. While death rates from smoking have declined in recent decades, the World Health Organization (WHO) reports that 15% of global deaths are attributable to smoking, with 1 in 7 deaths linked to cigarette smoking, particularly prevent among adults over 70 years old globally [1]. Additionally, WHO has identified a decrease in Global Smoking Prevalence (GSP) in High-Income Countries (HIC) and Upper-Middle-Income Countries (UMIC), while the number of smoking-related deaths has increased in Lower-Middle Income Countries (LMIC) and Low-Income Countries (LIC). Notable, Nauru, Kiribati, and Tuvalu are identified as the countries with the highest number of active smokers, respectively. Therefore, this study aims to validate the accuracy of the above statement with statistical evidence.

Stroke is the leading cause of disability and the second most ruthless cause of death globally [2]. When comparing global stroke statistics, stroke incidence has increased by 70%, death rates attributable to stroke by 43%, stroke prevalence by 102%, disability-adjusted life years by 143%, with a 50% lifetime stroke risk [3]. It is evident that, according to WHO, the countries included in LMIC and LIC have undergone a noticeable change in the values of SDR.

The relationship between stroke and smoking regarding risk rates and global mortality rates has been examined previously [4]. Past literature discusses a significant change in stroke death due to shifts in death rates attributable to smoking, and alterations in stroke incidence due to changes in GSP. However, the impact of GSP on global SDR has not been addressed in previous studies. This study explores the impact of Global Smoking Prevalence (GSP) on stroke incidence and mortality rates from 1990 to 2019 by incorporating polynomial regression analysis for time series and cross-sectional data. It collaborates with two global statistical databases, focusing on 27 selected countries to provide a better understanding of smoking prevalence and stroke. It analyses GSP and SDR rates for 27 countries closely with respect to their income groups (HIC, UMIC, LMIC, LIC) as classified by the World Bank. Moreover, it discusses how GSP influences SDR across different countries while illustrating the visualised comparison of the selected countries’ changes in GSP and SDR from period one (1990–1999) to period two (2010–2019). Finally, the study presents both pictorial and numerical representations of polynomial regression results, facilitating an easy analysis in variations of the finally selected 17 countries.

Past studies have incorporated various stroke-related theories, such as the Cascade theory [5], the Inflammation and Immune Response theory [6], and the Vascular theory [7]. According to the Cascade theory, an ischemic cascade is a series of pathophysiological events triggered by an increasing myocardial oxygen supply-demand imbalance, resulting in a continuous cycle of deterioration [8]. The inflammation and immune response theory elucidates the mechanism of the immune system’s mechanism in response to harmful stimuli like damaged cells, pathogens, toxic compounds, or irradiations [9]. The vascular theory suggests a sudden interruption of blood circulation to parts of the brain, encompassing both ischemic and hemorrhagic stroke [10, 11]. In Ischemic stroke, when the blood supply to parts of the brain is interrupted, the theory implies that stroke can occur due to blockages caused by factors affecting atherosclerosis, thrombosis, embolism, or blood clotting. Considering these theories, it is evident that smoking increases stroke risk due to blood vessel damage, elevated blood pressure, and blood clot formation, thereby heightening the global risk of stroke.

The burden of smoking, the causes of smoking prevalence, and the incidence of smoking-related deaths under different health conditions for other worldwide population parameters have been discussed in previous studies [12–14]. Some of these studies have concluded that the risk of stroke is remarkably high in smokers. A study in Kuwait related to the adult population established smoking as a recognised cause of cancer, lung cancer, coronary heart disease, and stroke in 1996 [15]. Additionally, the relationship between cigarette smoking and stroke has been previously studied, considering various factors in a few HIC and UMIC including China, USA, Hong Kong, Japan, Sweden, Norway, and New Zealand [4].

Moreover, the incidence of stroke or the burden of stroke incorporating cigarette smoking as an associated risk factor has been debated in numerous previous research studies [13, 16–18]. In most of these studies, hypertension, diabetes, smoking, and high cholesterol levels are considered significant risk factors associated with stroke. Additionally, a meta-analysis has been conducted to determine the association between secondhand smoking and stroke [19]. The study concluded that exposure to smoking can strongly associated with the risk of stroke incidence. A survey of data collected in 2022 states that hypertension and smoking remain commonly reported risk factors for stroke in the Philippines [2]. It is also observed that despite the heavy burden of stroke, appropriate interventions could reduce the risk of stroke in Kenya. Moreover, the same study recommends promoting a healthy diet, regular physical activities and smoking cessation to reduce the risk of stroke [20].

Furthermore, a relationship between stroke death and cigarette smoking is evident in many past studies. Accordingly to research findings, an overall 94% believe that smoking can be attributed to severe illnesses, while 75.5% think it will lead to stroke [21]. A study conducted in 2018, found that Japan and Singapore have the lowest smoking rates, followed by Bangladesh, Papua New Guinea, and Bhutan. Conversely, Mongolia and Indonesia have recorded the highest rates of stroke death [22]. The study also reports the SDR of Kuwait (10.02%), Mongolia (18.09%) and Uzbekistan (9.85%) for the year 2017. Furthermore, according to data reports from Kenya for 2018, 55.6% of all stroke cases were ischemic stroke, of which 16.1% were attributed to smoking [23]. Consequently, the incidence of stroke due to cigarette smoking is primarily observed in LMICs and next in LICs.

Considering the number of death rates related to stroke and smoking prevalence, many studies have noted specific figures for different countries around the globe. Countries such as, Japan, Singapore, Bangladesh, Papua New Guinea, Bhutan, Kuwait, Mongolia, Uzbekistan, and Kenya show supporting statistics illustrating a relationship between GSP and SDR variables.

## Methods

### Study design

This section presents observations obtained for 27 countries categorised into income groups, aver a 30-year period from 1990 to 2019. The study focuses on identifying whether there is any influence of GSP on the increase of SDR. Mean values of SDR for two periods, namely period one (1990–1999) and period two (2000–2019), were compared. Twenty-seven countries were identified as those showing an increase in the mean values of SDR. Therefore, countries that showed a decrease in SDR throughout the two periods were excluded, and only countries with an increase were selected for the study. This selection criteria were implemented to prioritize countries where an increase in SDR is significant because an increase in smoking death rates within a country poses a grave public health concern, leading to increased healthcare burdens. Additionally, the rise in SDR on a per capita basis underscores the urgent need for targeted interventions and policies to address this escalating public health crisis. The study incorporates GSP as the independent variable and SDR as the dependent variable, respectively. The data used throughout the study is presented in S1 Appendix.

### Information sources

This research study has utilised two databases to collect secondary health outcome data for two variables. The data utilised throughout the study is outlined in Table 1.

**Table 1:** Data sources and variables

Variable	Definitions	Measurement	Source
GSP	Global Smoking Prevalence	Per 100,000 people	IHME, Global Burden of Disease (2019) https://ghdx.healthdata.org/gbd-2019
SDR	Strokes Deaths	Per 100,000 people	Our World in Data database (2021) http://ghdx.healthdata.org/gbd-results-tool

Source: Compiled by the authors

### Statistical analysis

To analyse the impact of GSP on SDR, a unidirectional non-linear regression analysis model was incorporated to gain an in-depth insight into the relationship and impact between SDR and GSP. A second-order polynomial regression model was utilized for the statistical analysis phase of the study. The formulas used for the regression analysis are as follows,

1 $$ {SDR}_{it}={\alpha }_{0}+{\alpha }_{1}{GSP}_{it}+{\alpha }_{2}{GSP}_{it}^{2}+{\epsilon }_{it}$$

In Eq. (1) ${\alpha }_{0}$ denotes the change in stroke death rates when GSP is zero per 100 000 people, while${ \alpha }_{1}$ depicts the change in the death rates related to stroke when GSP increases by 1 unit per 100,000 people. Similarly, ${\alpha }_{2}$ represents the change in SDR when GSP^2^ is increased by 1 per 100,000 people, respectively.

In the captured equation, the cross-sectional (income group) data relating to the dependent and independent variables are denoted by the *i*^th^ term while *t* denotes the time series analysis measured by years. A panel dataset of the selected 204 countries for the 30 years was incorporated to run a panel regression, and the results obtained are attached in the S4 appendix. When dealing with nonlinear connections in research investigations, polynomial regression has been shown to be superior to basic linear regression. It enables capturing complicated high-order patterns and inflection points in the data, resulting in increased model fit. However, when polynomial regression is used in research, care is taken to avoid overfitting, maintain interpretability, and ensure appropriate data quality to forecast or predict with higher accuracy. Furthermore, to identify whether the panel dataset used for the study is stationary or not, a Levin-Lin-Chu test model was incorporated. According to the results, the panel dataset used for the study is stationary, and the results obtained are attached in the S5 appendix for further reference.

## Results

The data collected for the two variables, GSP and SDR, were analysed to observe the trends, changes, and regression effects over 30 years for 204 countries. After the analysis, 27 countries were selected to continue the study comprehensively. The results obtained in the study are discussed in this section.

**Fig. 1:**
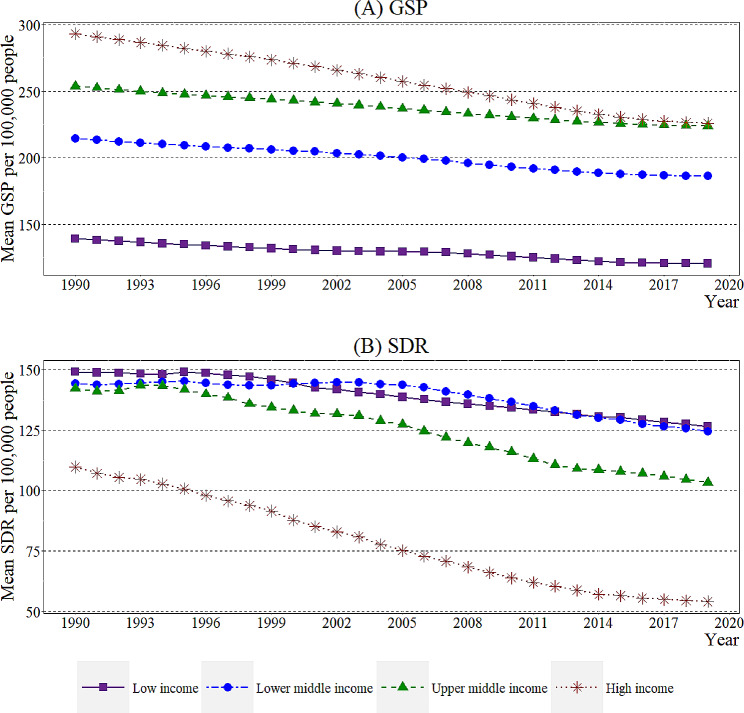
Incomegroup wise GSP and SDR from 1990–2019 Source: Authors’ illustrations based on data

According to data collected related to GSP and SDR, Fig. 1 depicts the mean rate of GSP and stroke death per 100,000 people separately from 1990 to 2019, clustered into four major income groups. The descriptive statistics for the selected countries clustered by income groups are portrayed in S2 Appendix. According to Fig. 1A, the mean values of GSP indicate that HIC resulted in the highest rates, while LC had the lowest mean values initially in 1990 compared to the other income groups. Although the HIC stratum has the highest GSP mean values throughout the period, unlike the other income groups, it shows a gradually declining trend. The LIC stratum remains the income group with the lowest GSP mean values until 2019, with a slight decline in the trend line throughout the period. Notably, there is decrease in all income groups from 2005. Considering the average decline income group-wise, an average drop of 2.344 in HIC, 1.077 in UMIC, 0.920 in LMIC, and 0.642 in LIC per 100,000 people throughout the period can be seen.

When considering the trends illustrated for the mean SDR from 1990 to 2019 categorised under the four major income bands, the HIC depicts a comparatively lower mean value of 52.946 death rates per 100,000 people for the variable compared with the other three income groups. In HIC, an average drop of 1.935 per 100,000 people in mean SDR values can be identified. The LIC stratum initially recorded the highest value of mean SDR in 1990, and the same position was recorded by the end of 2019. Apart from that, the LMIC group visualises fluctuations throughout the period. From 1993 to 1995 and 1999 to 2002, an increase in mean SDR can be seen. Altogether, from 1993 to 2005, various fluctuations in the trend lines of each income stratum can be identified. In contrast, after 2005, a significant decrease in the mean values of SDR for all income groups can be identified.

**Fig. 2:**
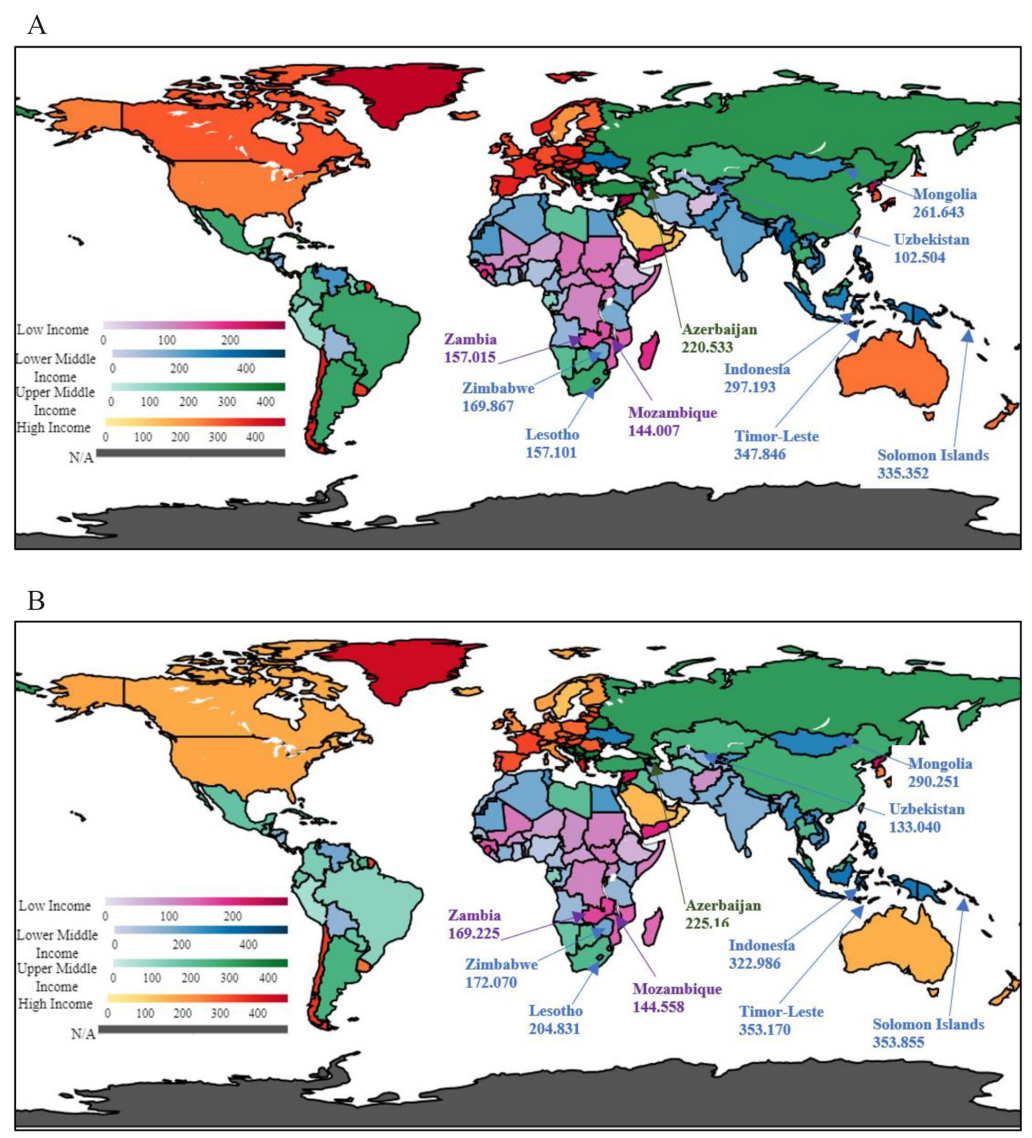
Average change in global smoking prevalence between two time periods from 1990–1999 and 2010–2019. **A:** Average mean values of GSP for the period 1990–1999. **B:** Average mean values of GSP for the period 2010–2019 Source: Authors’ illustrations based on data

Choropleth maps visually represent the average GSP for 1990–1999 and 2010–2019 in Fig. 2. After comparing the averages of the first period from 1990 to 1999 with the second period from 2010 to 2019, the countries considered for the study with an increase are plotted in the maps. Figure 2A illustrates the nations’ average number of GSPs from 1990 to 1999, which has shown a boost by the end of the second period, and Fig. 2B represents the average GSPs from 2010 to 2019 for the same countries. A total of 10 countries were identified including Azerbaijan, Mozambique, Zambia, Indonesia, Lesotho, Mongolia, Solomon Islands, Timor-Leste, Uzbekistan, and Zimbabwe, which show an increase in figures when compared to the mean values of the latest decade with the first decade of years of GSP for all 27 countries. If analysed income group-wise, out of 27, seven countries were included in the LMIC stratum. Therefore, when comparing all four income groups, the LMIC stratum has the highest number of countries with a significant positive impact.

The income stratum with the lowest number of countries having an increase is the UMIC stratum, where only Uzbekistan shows any change. Most importantly, none of the countries considered in the study that fall under the HIC stratum showed any increase in GSP when the two periods, 1990–1999 and 2010–2019, were compared. Apart from several countries, the highest mean increase of GSP when compared to the figures between two periods, Lesotho, which is from the LMIC, reports a rise of 47.7296 units per 100,000 people.

**Fig. 3:**
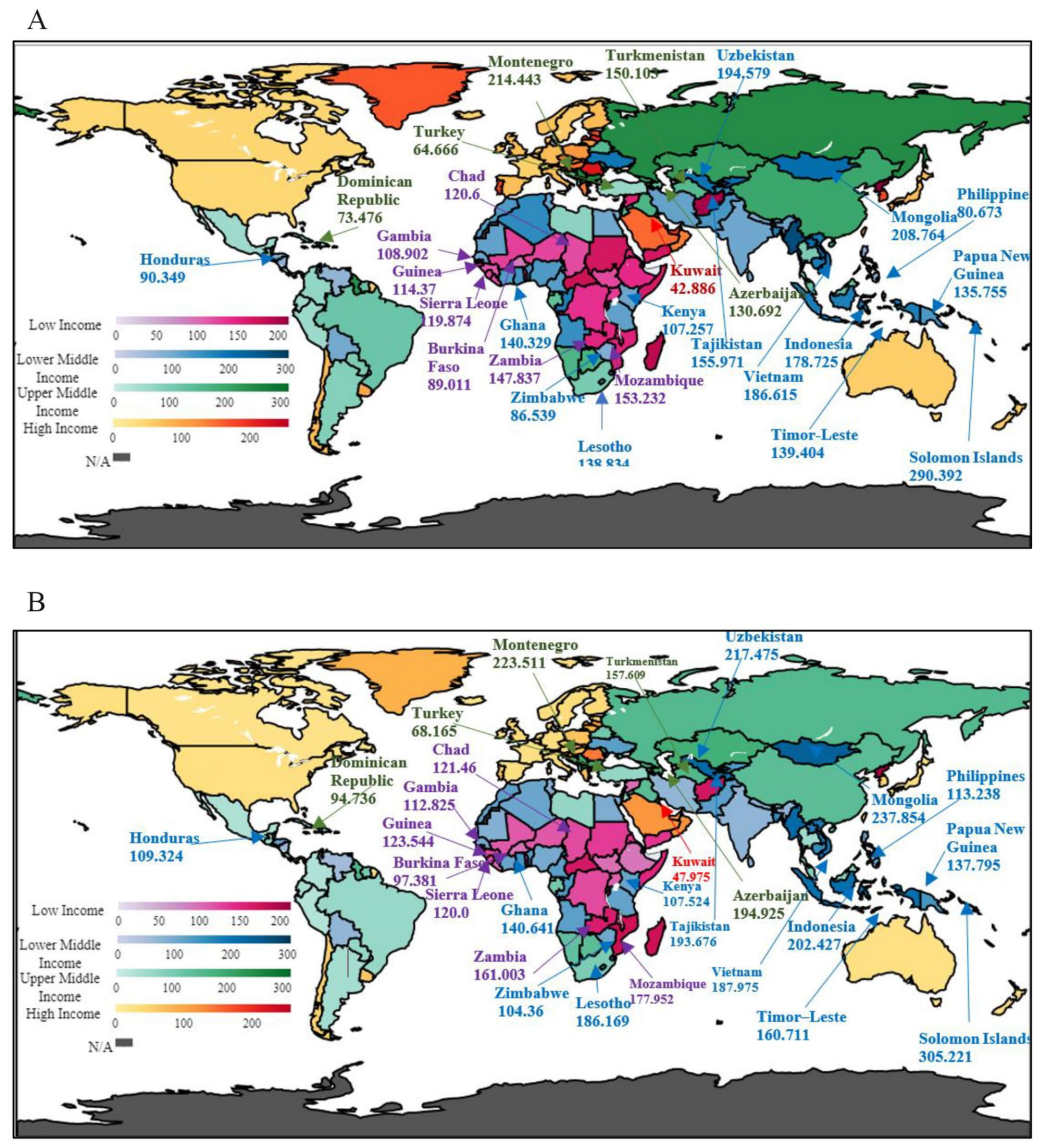
Average change in SDR between two time periods from 1990–1999 and 2010–2019. **A:** Average mean values of SDR for the period 1990–1999. **B:** Average mean values of SDR for the period 2010–2019 Source: Authors’ illustrations based on data

The average figures of stroke death are visualised in the above choropleth maps as provided in Fig. 3 for considering four major income groups. Figure 3A represents the intermediate units per 100,000 people from 1990 to 1999, and Fig. 3B illustrates the average units of stroke death per 100,000 people from 2010 to 2019. When comparing the changes in the two figures an overall increase in stroke death in the latest data can be seen in 27 countries. The most significant positive change in stroke death can be observed in Azerbaijan, with an increase of 64.233 death rates per 100,000 people compared to the values of Fig. 3A and B, where Sierra Leone has the lowest positive change of 0.191 death rates per 100,000 people. Overall, about nine countries, namely Dominican Republic, Indonesia, Lesotho, Mongolia, Mozambique, Philippines, Tajikistan, Timor-Leste, and Uzbekistan, show an increase of more than 20 units for the period 2010–2019 when compared with mean values of the period 1990–1999. Accordingly, when analysing the data of the periods, over 13% of the considered countries indicated an increase in stroke death by the end of 2019.

### Outcomes produced by the polynomial regression model

**Fig. 4:**
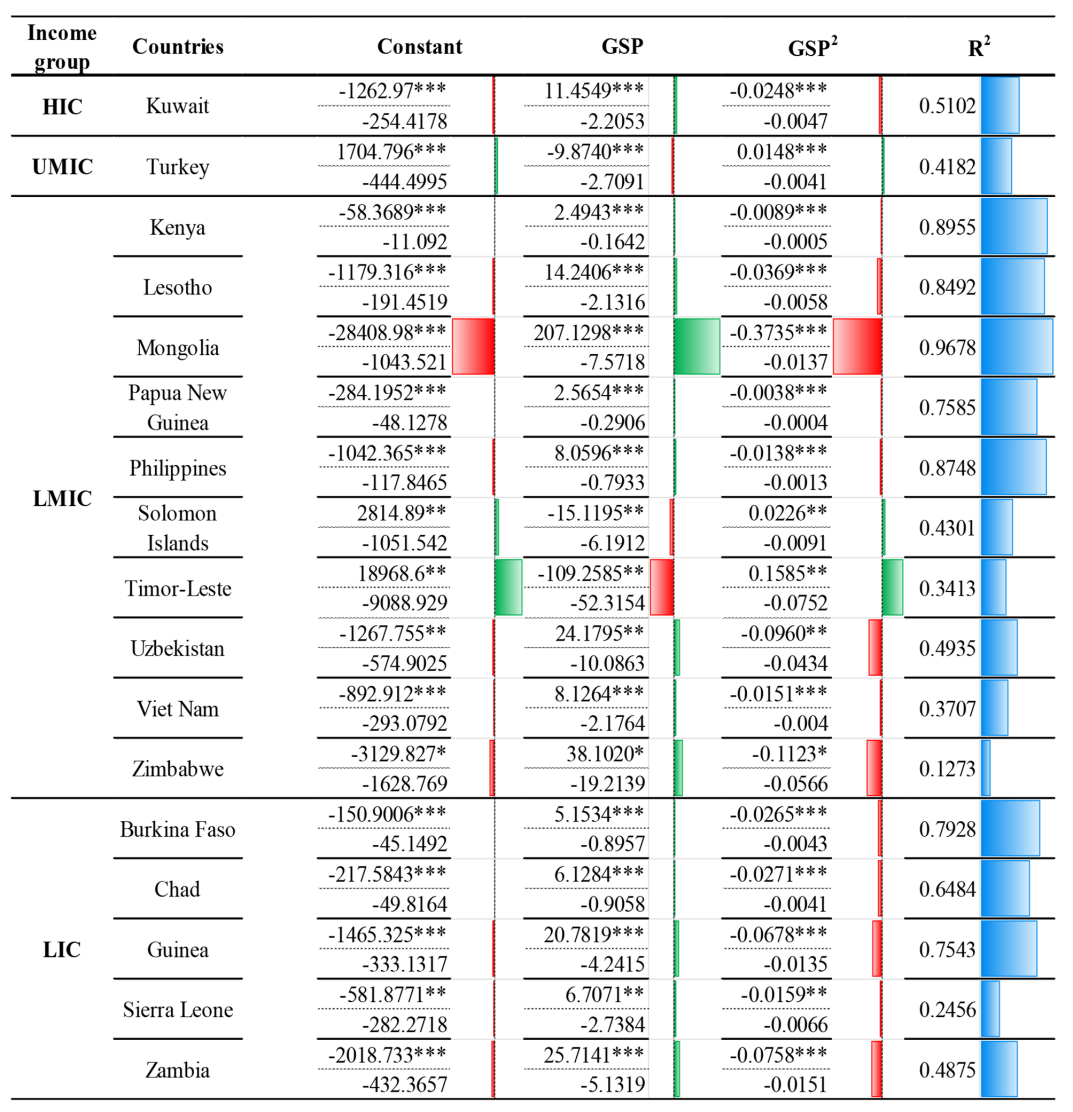
Polynomial regression results for the dependent variable SDR for selected countries Note: The asterisks *, ** and *** indicate 10%, 5% and 1% significance levels, respectively, robust standard errors are in parentheses

The polynomial regression was established to identify the non-linear relationship between SDR and GSP variables separately for the considered income groups: HIC, UMIC, LMIC and LIC countries, as presented in Fig. 4. The positive and negative impacts of SDR for the 27 countries are illustrated by the bars in green and red, with the R^2^ values presented in blue bars next to the particular value. Also, the size of the bars reflects the significance of SDR.

The polynomial regression analysis was applied to all 17 selected countries and further filtered after regressing based on the countries that show significance, as illustrated in Fig. 4. The descriptive statistics and polynomial regression results for the filtered 17 countries from the 27 significant countries are illustrated in the S3 Appendix. It can be observed that the polynomial results depict approximately 67 per cent of the 17 selected countries, offering value to GSP on SDR. Out of the 27 significant countries, countries such as Kuwait, Kenya, Lesotho, Mongolia, Papua New Guinea, the Philippines, Solomon Islands, Uzbekistan, Viet Nam, Zimbabwe, Burkina Faso, Chad, Guinea, Sierra Leone, and Zambia, have a positive impact. In Turkey, Solomon Islands and Timor-Leste, adverse effects throughout the period can be observed.

When comparing the values of the lower tercile, out of all the 17 countries, a positive impact can be observed in all countries except Turkey, Solomon Islands and Timor-Leste. Further, it can be observed that the highest positive effect of GSP on SDR can be seen in Mongolia with 207.129 units, secondly in Zimbabwe with 38.1020 units and thirdly in Zambia with 25.714 units per change in one unit of GSP per 100,000 people. Moreover, countries like Kenya, Burkina Faso, Papua New Guinea and Chad have the most negligible positive impact of GSP. In contrast, per change of one unit of GSP, Kenya shows an increase of 2 units, Papua New Guinea offers a rise of 3 units, Burkina Faso shows an increase of 5 units, and Chad shows an increase of 6 units of SDR per 100,000 people.

When considering the negative trends of GSP on SDR for the period, it can be seen that 17% of the countries show a negative impact of GSP on SDR per change of 1 unit of GSP per 100,000 people. As per the results of the significant countries, Turkey, Solomon Islands, and Timor-Leste show a negative impact of GSP on SDR, represented in the red bar. And it can be said that out of all the 17 significant countries, the three nations can be identified with a negative impact.

Accordingly, when compared with all income group strata, the LMIC stratum results in the highest number of countries that show a significant impact of GSP; most importantly, from the 14 countries with a significant positive effect on SDR, eight countries fall under the LMIC stratum. As for the adverse effects, the country with the highest negative impact on SDR also falls under this income stratum. Similarly, the government has the highest negative significant impact, and a positive significant effect on SDR falls under the LMIC income stratum.

## Discussion

This study aimed to assess the impact of GSP on SDR across the four major income strata identified for 204 countries from 1990 to 2019. Accordingly, as per the findings, it is evident that the independent variable GSP impacts SDR.

This study revealed that 62% of the 27 countries had significant impact on SDR. Several studies have illustrated country-specific evidence to identify the relationship between the two variables considering different dependent conditions like smoking status, socioeconomic status, age, gender and student attitudes towards smoking [24–26]. Those studies have incorporated countries like Hawaii, the United Arab Emirates, Kuwait, the US, Russia, and Denmark as the selected population of the study and have found that smoking can be a heterogeneous risk factor for the incidence of stroke in two ways: first, by directly causing damage to both the vascular system and its function. Second, smoking influences hemodynamic factors within the circulation.

The findings of the study further illustrate an increase in GSP in LMIC such as Lebanon, Iran, Egypt, Cote d’Ivoire, Congo, Bolivia, El Salvador, Djibouti, Timor-Leste, Kiribati, Solomon Islands, Indonesia, and Kyrgyzstan, and a few more countries from the LIC stratum, including Niger, Guinea Bissau, Zambia, Afghanistan, Mozambique, and the Democratic Republic of Congo. Most research studies on the LMIC and LIC strata have also observed smoking as a modifiable mortality risk over the two periods considered in Fig. 3A and B; countries such as Zambia, Mozambique, Timor-Leste, Indonesia, and the Solomon Islands show a positive significant impact of GSP on SDR in the considered two periods.

Furthermore, per the study’s findings from HIC, the stratum only in Kuwait shows a significant positive impact of GSP on SDR. As per past research done in 2019 across the globe, the statistics provide supporting information to prove that Stoke death rates in the HIC stratum and UMIC stratum are considerably lower where the study also refers to smoking as a modifiable risk of stroke mortality [27]. Accordingly, it is visible that a percentage of the countries that show a significant impact on SDR have negatively impacted SDR due to a change in one unit of GSP. Consequently, Turkey from UMIC, Solomon Islands and Timor-Leste from LMIC recorded a decrease in SDR per change of one unit in GSP. The Oceanian study also adds up evidence that even the county Kiribati, which is known to have the highest number of people who engage in cigarette smoking, found to have backup information to attest that smoking is a risk factor that contributes to the increase of stroke incidence [28].

Additionally, more past studies were conducted, including smoking prevalence as a preventable risk factor affecting stroke mortality and smoking cessation across the four major income groups: HIC, UMIC, LMIC, and LIC, respectively [29]. Respectively, the current study’s findings provide evidence to support that the impact of GSP has heavily affected countries classified under the LMIC stratum, and the second highest impact was identified on countries categorised under LIC. Many studies have identified these income groups as considerable variables in the studies based on different geographical locations [27, 30–33].

Finally, the study concludes that it is evident that the most negligible impact of smoking has occurred in developed countries, which are categorised under the HIC and UMIC strata. Accordingly, the study suggests that most countries classified as developing countries with lesser income levels smoke more cigarettes than developed countries. Eventually, they have an increased risk of stroke prevalence and incidence compared to developed countries with higher income levels.

## Conclusion

Smoking is stand as a significant risk factor contributing to global mortality, with stroke being a leading cause of disability and the second most severe cause of death globally. This study focused on the impact of GSP on SDR from 1990 to 2019 across four major income groups.

The LMIC and LIC strata displayed fluctuating behaviours in both SDR and GSP. When comparing average rates of GSP with SDR from period 1 (1990 to 1999) to period 2 (2010 to 2019), 27 countries showed an increase with the highest number falling under the LMIC stratum. Except for Turkey, Solomon Islands, and Timor-Leste form UMIC and LMIC, demonstrated a substantial negative effect. In contrast, 14 other countries had a positive impact of GSP on SDR. Mongolia reported the highest positive impact, with an increase of 207 units per change in 1 unit of GSP, while Timor-Leste showed the highest adverse impact, with a decrease of 109 units of SDR per change of 1 unit in GSP per 100,000 people.

Therefore, a clear relationship between GSP and SDR exists. Factors such age, gender, residence, wealth index, and education levels influence smoking prevalence, with hypertension, diabetes, cholesterol, and high levels of cigarette smoking directly impacting stroke death. LMICs demonstrate a higher impact of GSP on SDR, followed by countries in the UMIC stratum.

Even though the country with highest income levels illustrate higher levels of GSP and lower levels of SDR, LMIC and LIC strata show a higher significant impact of GSP on SDR than HIC and UMIC countries. Hence, countries with weaker economic statuses are more likely to smoke, leading to a higher incidence of stroke deaths.

### Limitations

While this study provides significant insights, there are several limitations to consider. First, the absence of data and statistics for the years 2020, 2021, 2022, and 2023 due to the unavailability of secondary data during the research process restricts the study’s scope and timeliness. Additionally, the inability to filter and identify deaths attributable to smoking separately from the SDR dataset for the considered period hampers a more nuanced analysis of the impact of smoking on stroke death rates. However, despite these limitations, the study focuses on the impact of GSP on SDR across four major income groups: HIC, UMIC, LMIC, and LIC from 1990 to 2019. Future studies could benefit from incorporating the latest statistics and employing more advanced analytical approaches to provide a more comprehensive understanding of the relationship between smoking prevalence and stroke death rates.

### Policy implications

The study underscores the importance of addressing smoking as a modifiable risk factor for stroke death, particularly in countries with varying economic statuses. While countries with robust economies and higher individual incomes tend to have higher cigarette consumption rates, they exhibit significantly lower stroke death rate. Conversely, countries with lower economic levels, such as LMICs and LICs, are less likely to engage in cigarette smoking but face a higher risk of stroke incidence and death.

The findings of the study highlight the urgent need for policymakers in LMICs and LICs to take proactive measures to combat the impact of smoking on stroke death rates. Implementing effective smoke cessation policies and programs can play a crucial role in preventing stroke deaths attributable to smoking. Additionally, promoting healthy diets and regular physical activity alongside smoking prevention and stroke death prevention initiatives can significantly enhance the quality of life and reduce the burden of stroke-related mortality.

By prioritising smoke cessation efforts and adopting comprehensive strategies that address multiple risk factors, policymakers can mitigate the impact of smoking on stroke death rates and improve public health outcomes in their respective countries.

### Electronic supplementary material

Below is the link to the electronic supplementary material.

Supplementary Material 1

Supplementary Material 2

Supplementary Material 3

Supplementary Material 4

Supplementary Material 5

## Data Availability

The datasets generated and analysed during the current study are publicly available at Our World in Data database: https://ourworldindata.org/ and Institute for Health Metrics and Evaluation (IHME) database: https://www.healthdata.org/
