# Supplementary material for: Examining the influence of global smoking prevalence on stroke mortality: insights from 27 countries across income strata
Source: BMC Public Health. 2024 Mar 19;24:857. doi: 10.1186/s12889-024-18250-1 (PMC10953178; doi:10.1186/s12889-024-18250-1)
Supplement: Supplementary file 2 — Supplementary Material 2 [file 12889_2024_18250_MOESM2_ESM.docx]

# S2 Appendix. Average positive change in SDR between 1990 – 1995 & 2015 - 2019 time periods.

| **Country** |  | **Income Group** | **Avg of**  **1990 - 1999** |  | **Avg of**  **2010 - 2019** |  | **Avg increase** |
| --- | --- | --- | --- | --- | --- | --- | --- |
| 1. Kuwait |  | **HIC** | 42.886 |  | 47.975 |  | 5.089 |
| 1. Azerbaijan |  | **UMIC** | 130.692 |  | 194.925 |  | 64.233 |
| 1. Dominican Republic |  |  | 73.476 |  | 94.736 |  | 21.26 |
| 1. Montenegro |  |  | 214.443 |  | 223.511 |  | 9.068 |
| 1. Turkey |  |  | 64.666 |  | 68.165 |  | 3.499 |
| 1. Turkmenistan |  |  | 150.103 |  | 157.609 |  | 7.506 |
| 1. Ghana |  | **LMIC** | 140.329 |  | 140.641 |  | 0.312 |
| 1. Honduras |  |  | 90.349 |  | 109.324 |  | 18.978 |
| 1. Indonesia |  |  | 178.725 |  | 202.427 |  | 23.702 |
| 1. Kenya |  |  | 107.257 |  | 107.524 |  | 0.267 |
| 1. Lesotho |  |  | 138.834 |  | 186.169 |  | 47.335 |
| 1. Mongolia |  |  | 208.764 |  | 237.854 |  | 29.09 |
| 1. Papua New Guinea |  |  | 135.755 |  | 137.795 |  | 2.04 |
| 1. Philippines |  |  | 80.673 |  | 113.238 |  | 32.565 |
| 1. Solomon Islands |  |  | 290.392 |  | 305.221 |  | 14.829 |
| 1. Tajikistan |  |  | 155.971 |  | 193.676 |  | 37.705 |
| 1. Timor-Leste |  |  | 139.404 |  | 160.711 |  | 21.307 |
| 1. Uzbekistan |  |  | 194.579 |  | 217.475 |  | 22.896 |
| 1. Viet Nam |  |  | 186.615 |  | 187.975 |  | 1.36 |
| 1. Zimbabwe |  |  | 86.539 |  | 104.36 |  | 17.821 |
| 1. Burkina Faso |  | **LIC** | 89.011 |  | 97.381 |  | 8.37 |
| 1. Chad |  |  | 120.621 |  | 121.46 |  | 0.839 |
| 1. Gambia |  |  | 108.902 |  | 112.825 |  | 3.923 |
| 1. Guinea |  |  | 114.372 |  | 123.544 |  | 9.172 |
| 1. Mozambique |  |  | 153.232 |  | 177.952 |  | 24.72 |
| 1. Sierra Leone |  |  | 199.874 |  | 120.065 |  | 0.919 |
| 1. Zambia |  |  | 147.837 |  | 161.003 |  | 13.166 |
