# Supplementary material for: Examining the influence of global smoking prevalence on stroke mortality: insights from 27 countries across income strata
Source: BMC Public Health. 2024 Mar 19;24:857. doi: 10.1186/s12889-024-18250-1 (PMC10953178; doi:10.1186/s12889-024-18250-1)
Supplement: Supplementary file 3 — Supplementary Material 3 [file 12889_2024_18250_MOESM3_ESM.docx]

## S3 Appendix. Summary descriptive statistics and Polynomial regression analysis results.

**Summary descriptive statistics for GSP and SDR of selected countries clustered by income group**

## Table s3.a: Descriptive statistics of HIC

| **Country** | **Variables** |  | **Mean** |  | **SD** |  | **Min** |  | **Max** |
| --- | --- | --- | --- | --- | --- | --- | --- | --- | --- |
| Kuwait | SDR |  | 49.46333 |  | 8.880813 |  | 39.39 |  | 72.23 |
|  | GSP |  | 237.0707 |  | 18.57254 |  | 202.9879 |  | 260.2065 |

Note: 30 observations have been considered for the above measures.

## Table s3.b: Descriptive statistics of UMIC

| **Country** | **Variables** |  | **Mean** |  | **SD** |  | **Min** |  | **Max** |
| --- | --- | --- | --- | --- | --- | --- | --- | --- | --- |
| Azerbaijan | SDR |  | 167.8923 |  | 33.12615 |  | 120.44 |  | 213.41 |
|  | GSP |  | 226.2677 |  | 6.174398 |  | 217.7492 |  | 237.2927 |
| Dominican Republic | SDR |  | 82.97033 |  | 10.27904 |  | 68.58 |  | 101.34 |
|  | GSP |  | 134.9059 |  | 11.07235 |  | 114.7479 |  | 147.7857 |
| Montenegro | SDR |  | 223.2753 |  | 11.68681 |  | 193.89 |  | 236.66 |
|  | GSP |  | 383.8828 |  | 17.07733 |  | 349.6455 |  | 405.6849 |
| Turkey | SDR |  | 64.61633 |  | 6.986178 |  | 50.07 |  | 75.29 |
|  | GSP |  | 329.5495 |  | 20.58626 |  | 302.274 |  | 358.346 |
| Turkmenistan | SDR |  | 156.6013 |  | 14.67859 |  | 129.03 |  | 184.44 |
|  | GSP |  | 182.7177 |  | 27.67658 |  | 125.299 |  | 220.269 |

Note: 30 observations have been considered for the above measures.

## Table s3.c: Descriptive statistics of LMIC

| **Country** |  | **Variables** |  | **Mean** |  | **SD** |  | **Min** |  | **Max** |
| --- | --- | --- | --- | --- | --- | --- | --- | --- | --- | --- |
| Ghana |  | SDR |  | 141.8227 |  | 4.17728 |  | 134.53 |  | 148.57 |
|  |  | GSP |  | 60.553 |  | 1.65516 |  | 58.29337 |  | 65.48547 |
| Honduras |  | SDR |  | 99.55133 |  | 9.520483 |  | 80.3 |  | 114.79 |
|  |  | GSP |  | 155.8043 |  | 12.85073 |  | 140.8466 |  | 176.7842 |
| Indonesia |  | SDR |  | 191.6717 |  | 10.63644 |  | 177.12 |  | 205.26 |
|  |  | GSP |  | 316.3914 |  | 16.44832 |  | 284.5339 |  | 335.4983 |
| Kenya |  | SDR |  | 109.2283 |  | 3.456368 |  | 103.63 |  | 115.14 |
|  |  | GSP |  | 140.6756 |  | 24.99869 |  | 106.7642 |  | 169.8674 |
| Lesotho |  | SDR |  | 166.7873 |  | 22.15221 |  | 134.08 |  | 191.83 |
|  |  | GSP |  | 176.8045 |  | 22.0095 |  | 153.8326 |  | 212.1427 |
| Mongolia |  | SDR |  | 243.9993 |  | 36.63548 |  | 184.38 |  | 298.12 |
|  |  | GSP |  | 275.5097 |  | 12.49136 |  | 260.2696 |  | 292.1814 |
| Papua New Guinea |  | SDR |  | 137.6013 |  | 2.301627 |  | 134.68 |  | 141.46 |
|  |  | GSP |  | 341.9031 |  | 33.21018 |  | 294.2061 |  | 374.5878 |
| Philippines |  | SDR |  | 105.0447 |  | 18.85119 |  | 74.53 |  | 128.06 |
|  |  | GSP |  | 307.7986 |  | 39.00911 |  | 247.1591 |  | 353.3316 |
| Solomon Island |  | SDR |  | 298.5297 |  | 7.117105 |  | 286.78 |  | 310.23 |
|  |  | GSP |  | 338.8802 |  | 12.91949 |  | 322.4073 |  | 358.3623 |
| Tajikistan |  | SDR |  | 184.8777 |  | 24.8536 |  | 127.21 |  | 214.7 |
|  |  | GSP |  | 118.0227 |  | 23.52884 |  | 90.085 |  | 164.89 |
| Timor-Leste |  | SDR |  | 148.5367 |  | 9.658508 |  | 137.94 |  | 166.71 |
|  |  | GSP |  | 348.4231 |  | 5.06516 |  | 339.898 |  | 355.052 |
| Uzbekistan |  | SDR |  | 231.8157 |  | 46.21201 |  | 135.31 |  | 309.5 |
|  |  | GSP |  | 121.9607 |  | 15.18487 |  | 93.688 |  | 136.099 |
| Viet Nam |  | SDR |  | 190.8247 |  | 9.033656 |  | 170.28 |  | 204.93 |
|  |  | GSP |  | 270.4936 |  | 25.6456 |  | 239.619 |  | 303.457 |
| Zimbabwe |  | SDR |  | 97.76533 |  | 9.371525 |  | 81.29 |  | 112.46 |
|  |  | GSP |  | 168.4452 |  | 5.759865 |  | 161.162 |  | 181.278 |

Note: 30 observations have been considered for the above measures.

## Table s3.d: Descriptive statistics of LIC

| **Country** | **Variables** |  | **Mean** |  | **SD** |  | **Min** |  | **Max** |
| --- | --- | --- | --- | --- | --- | --- | --- | --- | --- |
| Burkina Faso | SDR |  | 94.33033 |  | 4.071056 |  | 87.75 |  | 98.43 |
|  | GSP |  | 104.9383 |  | 11.37964 |  | 86.75774 |  | 118.4823 |
| Chad | SDR |  | 122.9363 |  | 3.768578 |  | 116.38 |  | 128.51 |
|  | GSP |  | 116.7517 |  | 14.26444 |  | 93.04862 |  | 130.2053 |
| Gambia | SDR |  | 111.6023 |  | 3.527084 |  | 106.63 |  | 117.48 |
|  | GSP |  | 147.9072 |  | 23.68906 |  | 121.5517 |  | 188.8416 |
| Guinea | SDR |  | 119.1983 |  | 4.504365 |  | 113.37 |  | 125.6 |
|  | GSP |  | 158.2657 |  | 5.546961 |  | 146.975 |  | 166.0434 |
| Mozambique | SDR |  | 163.2673 |  | 11.99698 |  | 149.65 |  | 182.68 |
|  | GSP |  | 145.4042 |  | 3.613187 |  | 140.6812 |  | 152.3194 |
| Sierra Leone | SDR |  | 121.694 |  | 3.965791 |  | 113.81 |  | 127.89 |
|  | GSP |  | 206.3937 |  | 9.179912 |  | 191.729 |  | 226.7285 |
| Zambia | SDR |  | 153.621 |  | 7.139866 |  | 140.12 |  | 170.69 |
|  | GSP |  | 166.6255 |  | 9.671844 |  | 156.176 |  | 183.06 |

Note: 30 observations have been considered for the above measures.

## Polynomial regression analysis results

**Table s3.e: Regression Results for the Dependent Variable SDR for selected HI countries.**

| **Countries** | |  | **Constant** |  | **GSP** |  | **GSP^2^** |  | **R^2^** |
| --- | --- | --- | --- | --- | --- | --- | --- | --- | --- |
| Kuwait | **SDR** |  | -1262.97*** |  | 11.4549*** |  | -0.0248*** |  | 0.5102 |
|  |  |  | (254.4178) |  | (2.2053) |  | (0.0047) |  |  |

Note: The asterisks, *, ** and *** indicate 10%, 5% and 1% significance levels, respectively. Robust standard errors in parentheses.

**Table s3.f: Regression Results for the Dependent Variable SDR for selected UMI countries.**

| **Countries** | |  |  | **Constant** |  | **GSP** |  | **GSP^2^** |  | **R^2^** |
| --- | --- | --- | --- | --- | --- | --- | --- | --- | --- | --- |
| Azerbaijan | **SDR** |  |  | -5765.54 |  | 48.8534 |  | -0.0999 |  | 0.4059 |
|  |  |  |  | (7797.548) |  | (68.5843) |  | (0.1507) |  |  |
| Dominican Republic | **SDR** |  |  | 288.5834 |  | -2.3110 |  | 0.0057 |  | 0.7155 |
|  |  |  |  | (187.7271) |  | (2.8651) |  | (0.0108) |  |  |
| Montenegro | **SDR** |  |  | 1025.762 |  | -4.6835 |  | 0.0067 |  | 0.4515 |
|  |  |  |  | (914.6457) |  | (4.8135) |  | (0.0063) |  |  |
| Turkey | **SDR** |  |  | 1704.796*** |  | -9.8740*** |  | 0.0148*** |  | 0.4182 |
|  |  |  |  | (444.4995) |  | (2.7091) |  | (0.0041) |  |  |
| Turkmenistan | **SDR** |  |  | -86.2936 |  | 3.0234** |  | -0.0090** |  | 0.2244 |
|  |  |  |  | (103.0459) |  | (1.2198) |  | (0.0035) |  |  |

Note: The asterisks, *, ** and *** indicate 10%, 5% and 1% significance levels, respectively. Robust standard errors in parentheses.

**Table s3.g: Regression Results for the Dependent Variable SDR for selected LMI countries.**

| **Countries** | |  | **Constant** |  | **GSP** |  | **GSP^2^** |  | **R^2^** |
| --- | --- | --- | --- | --- | --- | --- | --- | --- | --- |
| Ghana | **SDR** |  | -692.6549 |  | 25.3634 |  | -0.1912 |  | 0.6114 |
|  |  |  | (472.5291) |  | (15.4145) |  | (0.1256) |  |  |
| Honduras | **SDR** |  | -238.9575 |  | 4.9375 |  | -0.0176 |  | 0.6785 |
|  |  |  | (257.5476) |  | (3.3014) |  | (0.0105) |  |  |
| Indonesia | **SDR** |  | -57.0161 |  | 1.0622 |  | -0.0008 |  | 0.6461 |
|  |  |  | (511.8443) |  | (3.2927) |  | (0.0052) |  |  |
| Kenya | **SDR** |  | -58.3689*** |  | 2.4943*** |  | -0.0089*** |  | 0.8955 |
|  |  |  | (11.0920) |  | (0.1642) |  | (0.0005) |  |  |
| Lesotho | **SDR** |  | -1179.316*** |  | 14.2406*** |  | -0.0369*** |  | 0.8492 |
|  |  |  | (191.4519) |  | (2.1316) |  | (0.0058) |  |  |
| Mongolia | **SDR** |  | -28408.98*** |  | 207.1298*** |  | -0.3735*** |  | 0.9678 |
|  |  |  | (1043.521) |  | (7.5718) |  | (0.0137) |  |  |
| Papua New Guinea | **SDR** |  | -284.1952*** |  | 2.5654*** |  | -0.0038*** |  | 0.7585 |
|  |  |  | (48.1278) |  | (0.2906) |  | (0.0004) |  |  |
| Philippines | **SDR** |  | -1042.365*** |  | 8.0596*** |  | -0.0138*** |  | 0.8748 |
|  |  |  | (117.8465) |  | (0.7933) |  | (0.0013) |  |  |
| Solomon Islands | **SDR** |  | 2814.89** |  | -15.1195** |  | 0.0226** |  | 0.4301 |
|  |  |  | (1051.542) |  | (6.1912) |  | (0.0091) |  |  |
| Tajikistan | **SDR** |  | -77.1167 |  | 5.1762*** |  | -0.0241*** |  | 0.7774 |
|  |  |  | (66.1584) |  | (1.1014) |  | (0.0044) |  |  |
| Timor-Leste | **SDR** |  | 18968.6** |  | -109.2585** |  | 0.1585** |  | 0.3413 |
|  |  |  | (9088.929) |  | (52.3154) |  | (0.0752) |  |  |
| Uzbekistan | **SDR** |  | -1267.755** |  | 24.1795** |  | -0.0960** |  | 0.4935 |
|  |  |  | (574.9025) |  | (10.0863) |  | (0.0434) |  |  |
| Viet Nam | **SDR** |  | -892.912*** |  | 8.1264*** |  | -0.0151*** |  | 0.3707 |
|  |  |  | (293.0792) |  | (2.1764) |  | (0.0040) |  |  |
| Zimbabwe | **SDR** |  | -3129.827* |  | 38.1020* |  | -0.1123* |  | 0.1273 |
|  |  |  | (1628.769) |  | (19.2139) |  | (0.0566) |  |  |

Note: The asterisks, *, ** and *** indicate 10%, 5% and 1% significance levels, respectively. Robust standard errors in parentheses.

**Table s3.h: Regression Results for the Dependent Variable SDR for selected LI countries.**

| **Countries** | |  | | **Constant** | |  | | **GSP** | |  | | **GSP^2^** | |  | | **R^2^** | |  |
| --- | --- | --- | --- | --- | --- | --- | --- | --- | --- | --- | --- | --- | --- | --- | --- | --- | --- | --- |
| Burkina Faso | **SDR** | |  | | -150.9006*** | |  | | 5.1534*** | |  | | -0.0265*** | |  | | 0.7928 | |
|  |  |  |  | | (45.1492) | |  | | (0.8957) | |  | | (0.0043) | |  | |  |  |
| Chad | **SDR** | |  | | -217.5843*** | |  | | 6.1284*** | |  | | -0.0271*** | |  | | 0.6484 | |
|  |  |  |  | | (49.8164) | |  | | (0.9058) | |  | | (0.0041) | |  | |  |  |
| Gambia | **SDR** | |  | | 114.2963*** | |  | | 0.0500 | |  | | -0.0004 | |  | | 0.3450 | |
|  |  |  |  | | (30.5499) | |  | | (0.4090) | |  | | (0.0013) | |  | |  |  |
| Guinea | **SDR** | |  | | -1465.325*** | |  | | 20.7819*** | |  | | -0.0678*** | |  | | 0.7543 | |
|  |  |  |  | | (333.1317) | |  | | (4.2415) | |  | | (0.0135) | |  | |  |  |
| Mozambique | **SDR** | |  | | -4169.845 | |  | | 58.4885 | |  | | -0.1971 | |  | | 0.0865 | |
|  |  |  |  | | (4430.059) | |  | | (60.5811) | |  | | (0.2070) | |  | |  |  |
| Sierra Leone | **SDR** | |  | | -581.8771** | |  | | 6.7071** | |  | | -0.0159** | |  | | 0.2456 | |
|  |  |  |  | | (282.2718) | |  | | (2.7384) | |  | | (0.0066) | |  | |  |  |
| Zambia | **SDR** | |  | | -2018.733*** | |  | | 25.7141*** | |  | | -0.0758*** | |  | | 0.4875 | |
|  |  |  |  | | (432.3657) | |  | | (5.1319) | |  | | (0.0151) | |  | |  |  |

Note: The asterisks, *, ** and *** indicate 10%, 5% and 1% significance levels, respectively. Robust standard errors in parentheses.
