# Supplementary material for: Examining the influence of global smoking prevalence on stroke mortality: insights from 27 countries across income strata
Source: BMC Public Health. 2024 Mar 19;24:857. doi: 10.1186/s12889-024-18250-1 (PMC10953178; doi:10.1186/s12889-024-18250-1)
Supplement: Supplementary file 4 — Supplementary Material 4 [file 12889_2024_18250_MOESM4_ESM.docx]

| **Variable** | | **FE** | **RE** |
| --- | --- | --- | --- |
| GSP | | 0.314*** | 0.301*** |
|  |  | (0.009) | (0.009) |
| Constant | | 46.560*** | 49.472*** |
|  |  | (2.118) | (4.382) |
| R^2^ | Within | 0.1535 | |
|  | Between | 0.0167 | |
|  | Overall | 0.0223 | |
| No. of years/ No. of countries | | 30/204 | |
| N | | 6120 | |
| F-Test | | 282.92*** | |
| LM test | | 70971.27*** | |
| Hausman test | | 38.32*** | |

**S4 Appendix. Panel regression results of dependent variable SDR for 204 countries**

Note: The asterisks, *, ** and *** indicate 10%, 5% and 1% significance level, respectively. Robust standard errors in parentheses. FE and RE represent the Fixed Effect and Random Effect, respectively. The hypothesis for F-test is Ho: Accepting POLS and Ha: Accepting FE Model, the hypothesis for the Breusch-Pagan test is Ho: Accepting POLS and Ha: Accepting RE Model and the hypothesis for the Hausman test are Ho: Accepting RE Model and Ha: Accepting FE Model.
