# Supplementary material for: Examining the influence of global smoking prevalence on stroke mortality: insights from 27 countries across income strata
Source: BMC Public Health. 2024 Mar 19;24:857. doi: 10.1186/s12889-024-18250-1 (PMC10953178; doi:10.1186/s12889-024-18250-1)
Supplement: Supplementary file 5 — Supplementary Material 5 [file 12889_2024_18250_MOESM5_ESM.docx]

**S5 Appendix. Levin-Lin-Chu unit-root test results of SDR for 204 countries**

| Unadjusted t | -21.5034 |
| --- | --- |
| Adjusted t* | -15.2039*** |
| Number of countries | 204 |
| Number of years | 30 |

Note: Ho: panels contain unit roots

Ha: panels are stationary.

The asterisks, *, ** and *** indicate 10%, 5% and 1% significance level, respectively.
